# Tailoring the Functional Potential of Red Beet Purées by Inoculation with Lactic Acid Bacteria and Drying

**DOI:** 10.3390/foods9111611

**Published:** 2020-11-06

**Authors:** Gabriel-Dănuț Mocanu, Ana Cosmina Chirilă, Aida Mihaela Vasile, Doina Georgeta Andronoiu, Oana-Viorela Nistor, Vasilica Barbu, Nicoleta Stănciuc

**Affiliations:** Department of Food Science, Food Engineering, Biotechnology and Aquaculture, Faculty of Food Science and Engineering, “Dunarea de Jos” University of Galați, 800201 Galați, Romania; Danut.Mocanu@ugal.ro (G.-D.M.); cosminachana@gmail.com (A.C.C.); Aida.Vasile@ugal.ro (A.M.V.); Georgeta.Andronoiu@ugal.ro (D.G.A.); Oana.Nistor@ugal.ro (O.-V.N.); Vasilica.Barbu@ugal.ro (V.B.)

**Keywords:** beetroot, convective drying, infrared drying, purée, antioxidant activity, Fourier-transform infrared spectroscopy, confocal scanning microscopy, texture

## Abstract

This study was focused on a comparative analysis of two drying methods, such as convective and infrared drying, on the red beetroot purées with lactic acid bacteria, as a strategy for tailoring the health benefits of the selected plant. For both varieties, the total betalain contents varied from 13.95 ± 0.14 mg/g dry weight in *Beta vulgaris* var. *cylindra* when compared with 11.09 ± 0.03 mg/g dry weight in *Beta vulgaris* var. *vulgaris*, whereas significant differences were found in total phenolic and flavonoid contents. Significant drying induced changes were found in selected bioactives, in terms of total betalains, flavonoids, and polyphenols, which influenced the antioxidant activities of the purées, structure, and color parameters. In general, infrared technology was more protective, leading to an increase of 20% in flavonoids content. One logarithmic decrease in cell viability was observed in all powders samples. After the in vitro digestion, the betalains decreased, in both gastric and intestinal simulated juices, with a more pronounced profile in infrared processed purées. Textural and rheological analysis of the dried purées highlighted that the infrared drying is milder compared to the conventional one, allowing us to obtain powders with enhanced functional properties, in terms of bioactives content, cell viability, color, and structural and rheological behavior.

## 1. Introduction

Considered in recent years as key components providing substantial benefits to human health, biologically active compounds, such as polyphenols and betalains, have recently gained particular interest from both academia and relevant industry from the perspective of developing foods and ingredients with positive effects for health [1].

Red beet (*Beta vulgaris*) are among the most popular varieties of vegetables, not only because of its significant content in biologically active compounds, such as polyphenols and betalains, but also their intense aromatic flavor [2]. Betalains are known as water-soluble nitrogen-containing pigments, consisting of two sub-classes: betacyanins (red-violet pigments) and betaxanthins (yellow-orange pigments) [3]. The health benefits of betalains are described as antimicrobial [4], inhibition of cell proliferation of human tumor cells [5], prevention of diseases like cancer, cardiovascular diseases [6], anti-inflammatory effects, antiradical and antioxidant activity [7], and enriching human low-density lipoproteins, which increase resistance to oxidation [8].

It is well known that color is an important quality indicator that determines the consumers’ acceptance of foods. A more extensive application of natural colorants over synthetic ones has been observed recently on the market, especially because natural coloring is commonly associated with positive effects on human health and can also be used as functional additives. The use of pigments from a natural origin is usually limited due to their low stability, solubility, bioavailability, interaction with other components of the food matrix, etc. [9]. From the perspective of developing food products with enhanced properties, from both nutritional and functional point of view, it is necessary to choose appropriate methods for obtaining and stabilizing these valuable compounds.

Several colorants may be used for coloring foods, such as anthocyanins and carotenoids. The use of betalains for coloring food products is approved by the European Union and betalains are labeled as E-162; however, the use of betalains for specific uses provide several advantages, since these pigments, having the ability to cover a wide color range, from red to purple, are considered to be more stable to pH and temperature-induced changes. Due to the higher stability in a wider pH range, the use of betalains as a dye allows the replacement of anthocyanins for low-acid foods [3]. When comparing to carotenoids, which exhibit a yellow-orange color range, their applications are limited due to poor solubility in water; however, betaxanthins could be successfully used in application as yellow-orange food colorants [10].

Drying is a classical method of food preservation, providing several advantages such as smaller space for storage, lighter weight for transportation, and longer shelf-life [11]. Additionally, dried vegetables may be used as ingredients in ready-to-eat meals in order to add value by improving their functional quality due to health benefit compounds (vitamins, phytochemicals, and dietary fibers), and therefore, drying methods have acquired particular importance from the perspective of selecting methods that preserve the untainted qualities of the products [12]. One of the most used methods for drying vegetables and fruits is convective drying, which uses hot air to heat and remove water from the product [13].

The infrared assisted drying technology is one of the growingly popular approaches to provide heat for the drying of moist materials [14], allowing infrared radiation energy to be transmitted from the heating agent to the product. The technology brings some advantages, such as quick and homogeneous heating of the material without heating the surrounding air [15] where the generated heat in a layer below the surface is transferred to the material’s center and surface. Additionally, due to the moisture transfer from the material’s center to the surface, the heat and mass transfers are concurrent and countercurrent in layers, close to the material’s surface and its other parts, respectively [16].

The main objective of this study was the comparative analysis of two techniques for drying red beetroot purées, impregnated with lactic acid bacteria to obtain powders with functional potential. Two drying methods were used: convective and infrared assisted drying methods. The red beetroot purées were analyzed for betalains content, antioxidant activity, cell viability, color, and in vitro release of total betalains. The structural and morphological particularities of the powders after drying were analyzed by using confocal laser microscopy. The drying induced changes in powders were analyzed by Fourier transform infrared spectroscopy (FT-IR). Textural properties and non-linear viscoelastic behavior were also analyzed for the rheological characterization of the powders. 

## 2. Materials and Methods 

### 2.1. Plant Materials

Two types of fresh red beet (taproots of *Beta vulgaris* L. var. *vulgaris* and *Beta vulgaris* L. var. *cylindra*) were obtained from the local vegetable market in Galați, Romania respectively Cahul, Republic of Moldova and stored at 4 °C before the analysis (not exceeding 48 h from procurement). The initial moisture content of the samples was 88.05 ± 0.20% and 88.17 ± 0.40% for *Beta vulgaris* L. var. *vulgaris* and *Beta vulgaris* L. var. *cylindra*, respectively, while the final moisture content was 8.03 ± 0.30% and 8.12 ± 0.20%. 

### 2.2. Lactic Bacteria

The commercial culture *Lactobacillus casei* ssp. *paracasei* (*L. casei* 431^®^) was provided by Chr. Hansen (Hoersholm, Denmark).

### 2.3. Sample Preparation

#### 2.3.1. Inoculum Preparation

The freeze-dried strain was reactivated in MRS (de Man, Rogosa, and Sharpe agar) broth (Merk, Darmstadt, Germany), at 37 °C for 24 h and grown under these conditions until achieving high cell concentration (>10^9^ CFU/mL). The method described by Begot et al. [17] was used to estimate the bacterial cell concentration by the turbidimetric method. The logarithmic value of bacterial cell concentration was obtained by dilution and pour plate counting after incubation in MRS agar at 37 °C for 48 h under aerobic conditions [18].

#### 2.3.2. Red Beet Pureés Preparation

Fresh beetroots were washed, peeled, and cut into small pieces. The purée was prepared by blending 1 kg from each variety of fresh red beet with 87.5 g of double distilled water for 5 min using a kitchen blender Philips HR2100/40. An inoculum of 1% of *L. casei* 431^®^ was added into the purée samples. Six variants of red beet purée were obtained, coded as follows: BV_0_—fresh sample of *Beta vulgaris* L. var. *vulgaris*, BC_0_—fresh sample of *Beta vulgaris* L. var. *cylindra*, BV_C_, BC_C_—red beet purée dried by convection method, BV_IR_, BC_IR_—red beet purée dried by infra-red (IR) method.

### 2.4. Drying of the Samples

Drying experiments were carried out in an oven dryer with 5 perforated trays (mesh trays) (Concept SO4000 Infra 500W, Chocen, Czech Republic). The red beet purée samples were placed on a stainless steel tray with a surface area of 0.072 m^2^. To ensure the optimal parameters of the drying process, the dryer was preheated for 1 h before use. The red beet purée (approximately 350 g for each variant) was spread on the tray surface obtaining a 1.5 mm thick layer using a digital micrometer. The layer thickness was measured in five points of the baking paper (in the four corners and the middle). The drying experiment was performed at a constant air velocity of 1.1 m∙s^−1^, a relative humidity of 11.2%, and a drying temperature of 45 °C, while the ambient air temperature was 20 °C. The air velocity was measured with a VT 115 hotwire thermo-anemometer (Kimo Instruments, Millgrove, Ontario, Canada). The relative humidity was measured with a thermo hygrometer EE33 Series, fitted with a sensing probe (E + E Electronik Ges.m.b.H. Engerwitzdorf, Austria). The drying of red beet purée samples was carried out until the equilibrium humidity (3 h and 30 min in the case of convective drying and 2 h and 30 min for infrared drying). After each drying experiments, all the samples were cooled under laboratory conditions and stored in airtight containers. All drying experiments were performed in triplicate.

### 2.5. Extraction of Betalains

The extraction step of betalains was performed as described by Ravichandran et al. [19]. Briefly, 0.1 g of dried samples were mixed with 10 mL of 50% ethanol, agitated for 10 s, followed by centrifugation at 6000× *g* for 10 min. To increase the yield of betalains extraction, the supernatant was collected, and the extraction was repeated 3 more times. The supernatant was further used for the determination of betalains.

### 2.6. Determination of Betalain Compounds by Spectrophotometric Methods

The content of betaxanthins and betacyanins in the extracts was determined spectrophotometrically at 538 and 480 nm with a UV–Vis spectrometer.

The total betalain content (*BC*) was calculated using Equation (1):

(1) $$BC\;\left(\frac{mg}{g}\right)=\frac{A\cdot DF\cdot M_{w}\cdot 1000}{ex1}$$

where *A* is the absorption, *DF* the dilution factor, and l the pathlength (1 cm) of the cuvette. For quantification of betacyanins and betaxanthins, the molecular weights (*M_w_*) and molar extinction coefficients (*e*) (*M_w_* of 550 g/mol; *e* of 60,000 L/mol·cm in H_2_O) and (*M_w_* of 308 g/mol; *e* of 48,000 L/mol·cm in H_2_O) were applied.

### 2.7. Antioxidant Activity

The antioxidant activity of the ultrasound-assisted extracts dissolved in ethyl acetate was measured by using the modified ABTS (2,2′-Azino-bis(3-ethylbenzothiazoline-6-sulfonic acid) radical decolorization assay according to the method described by Miller and Rice—Evans [20]. The experiments were performed in triplicate.

### 2.8. Cells Viability

For viable cell counting of *L. casei* 431^®^, 10-fold serial dilutions of the samples were performed using sterile physiological serum (0.9 g NaCL%, *w*/*v*). The pour plate technique was employed. The viable cell number was determined by estimating the number of colony-forming units on the MRS-agar plates after incubation at 37 °C for 48 h. The counts were expressed as colony-forming units per gram. The viability of *L. casei* 431^®^ was determined immediately after manufacturing and during storage at 7, 14, and 21 days.

### 2.9. In Vitro Release of the Betalains

The protocol described by Oancea et al. [21] was used to perform red beetroot dried purées to a simulated in vitro gastrointestinal digestion process, mimicking gastric and intestinal phases. The post-hydrolysis fractions were collected at every 30 min of digestion, centrifuged (10,000× *g* for 10 min), and analyzed for betalains content.

### 2.10. Color Parameters

The color parameters of fresh and dried samples were performed using a MINOLTA Chroma Meter CR-410 (Konica Minolta, Osaka, Japan). For color analysis, it is necessary to have a homogeneous powder the dried samples were ground for 20 s with a grinder (Gorenje SMK150B, Velenje, Republic of Slovenia). To obtain fresh red beetroot purée, the red beet was blended at 1900 rpm for 3 min with a laboratory blender (Philips HR2100/40, EC) to achieve a uniform color. The color parameters determined for fresh and dried red beetroot were L* (lightness/darkness), a* (red/green), and b* (yellow/blue). The total color difference (Δ*E*) between samples was calculated according to Equation (2) [22]:

(2) $$\Delta E=\;\sqrt{{\left(L_{0}^{*}-\;L^{*}\right)}^{2}+{\left(a_{0}^{*}-a^{*}\right)}^{2}+{\left(b_{0}^{*}-b^{*}\right)}^{2}}$$

Subscript 0 refers to the color of the fresh sample. The color intensity (C*) and visual color appearance (h*) were calculated according to Equations (3) and (4) [22]:

(3) $$C^{*}=\sqrt{a^{*2}+b^{*2}}$$

(4) $$h^{*}=\;tan^{-1}\left(\frac{b^{*}}{a^{*}}\right)$$

L*, a*, b* values were used to establish the whiteness index according to the following Equation (5) [23]:

(5) $$WI=100-{\left[{\left(100-L^{*}\right)}^{2}+a^{*2}+b^{*2}\right]}^{1/2}$$

According to Maskan [24] the browning index (*BI*) and the yellowness index (*YI*) were calculated using Equations (6) and (7):

(6) $$BI=100\times \left(\frac{X-0.31}{0.17}\right)$$

where

(7) X=(a∗+1.75·L∗)(5.645·L∗+a∗−3.012·b^) and  YI= 142.86·b∗L∗

The color parameters are dimensionless. The color analysis was performed in triplicate.

### 2.11. Structural and Morphological Properties of the Dried Powders

To highlight the functional potential of red beet purées inoculated with *L. casei* 431 strain, the confocal laser scanning microscopy (CLSM) analysis was performed using an LSM 710 system (Carl Zeiss MicroImagining, Göttingen, Germany) equipped with a diode laser (405 nm), Ar-laser (458 nm, 488 nm, 514 nm), diode-pumped solid-state laser (DPSS; 561 nm) and HeNe-laser (633 nm). The 3D images were acquired with an AxioObserver Z1 inverted microscope (20x apochromatic objective, numerical aperture 1.4) and analyzed by ZEN 2012 SP1 software (black edition; Carl Zeiss MicroImagining, Göttingen, Germany). The autofluorescence of the samples was captured in the range of 500–660 nm wavelengths (emission spectrum of betalains) [25]. The acquisition parameters of the images were: mean method, line scan mode, speed 6, and 12-bit depth. In order to increase the signal-to-noise ratio, a frame average of eight scans was used.

### 2.12. FT-IR 

The infrared spectra were collected using a Nicolet iS50 FT-IR spectrometer (Thermo Scientific, Waltham, MA, USA) equipped with a built-in ATR (Attenuated Total Reflectance) accessory, DTGS (Deuterated Triglycine Sulfate) detector, and KBr beam splitter. 32 scans were co-added over the range of 4000–400 cm^−1^ with a resolution of 4 cm^−1^. Air was taken as the reference for the background spectrum before each sample. After each spectrum, the ATR plate was cleaned with ethanol solution. In order to verify that no residue from the previous sample remained, a background spectrum was collected each time and compared to the previous background spectrum. The FT-IR spectrometer was sited in a room that was air-conditioned with controlled temperature (21 °C).

### 2.13. Textural and Oscillatory Measurements 

#### 2.13.1. Texture Analysis 

Fresh and reconstituted beetroot purée samples were poured into cylindrical plastic containers with 40 mm diameter and 50 mm length so that the height of the samples was 40 mm. A double penetration test was applied, using a 25.4 mm acrylic cylinder of a Brookfield CT3 texture analyzer (AMETEK Brookfield, Middleboro, MA, USA). The testing parameters were set as follows: target distance 10 mm, trigger load 0.067 N, pretest speed 2 mm/s, test speed 1 mm/s, return speed 1 mm/s, and load cell 1000 g. The textural parameters (firmness, adhesiveness, cohesiveness, and springiness) were determined with TexturePro CT V1.5 software, provided by Brookfield Engineering Labs. Inc., (Middleborough, MA, USA). 

#### 2.13.2. Rheological Analysis 

The rheological analysis was performed with an AR2000ex controlled rheometer (TA Instruments, New Castle, DE, USA). Firstly, to identify the linear viscoelastic region, a strain sweep test was applied, maintaining a frequency of 1 Hz and varying the strain between 0.01 and 100%. Then, a dynamic frequency sweep test was applied, between 0.1 and 100 Hz, at a constant strain of 0.2 Hz. In all cases, storage modulus (G′) and loss modulus (G″) were registered. All the measurements were made at 25 °C, with a 40 mm diameter geometry and o closure gap of 2 mm. 

### 2.14. Statistical Analysis

All analyses were performed in triplicate and data reported as mean ± standard deviation (SD). To identify significant differences, experimental data were subjected to one-way analysis of variance (ANOVA) after running the normality and homoscedasticity tests. The Tukey method with a 95% confidence interval was employed for post-hoc analysis; *p* < 0.05 was considered to be statistically significant. The statistical analysis was carried out using Minitab 18 software.

## 3. Results and Discussion

### 3.1. The Content of Selected Phytochemicals in Fresh Red Beet Purées

Initially, the betalains content of the fresh red beet pureés was measured by using spectrophotometric methods. A significantly (*p* < 0.05) higher content of total betalains was found in BC_0_ pureés of 13.95 ± 0.14 mg/g DW when compared with 11.09 ± 0.03 mg/g DW in BV_0_. Both varieties showed (Table 1) a similar content in betaxanthins, of 4.71 ± 0.06 mg/g DW and 4.06 ± 0.01 mg/g DW, whereas significant differences (*p* < 0.05) were found in betacyanins, with 9.23 ± 0.07 mg/g DW and 7.03 ± 0.02 mg/g DW, respectively. The total polyphenolic content (TPC) showed significant differences (*p* < 0.05), with a more than two-twice higher concentration of total polyphenols in BC_0_ (71.94 ± 2.21 mg GAE/g DW) when compared with BV_0_ (32.88 ± 0.34 mg GAE/g DW). Total flavonoids were found in a higher concentration in BV_0_ of 36.00 ± 1.78 mg CE/g DW, when compared with 23.31 ± 1.34 mg CE/g DW in BC_0_. The antioxidant profile showed significant differences (*p* < 0.05), with values of 93.46 ± 2.51 mMol Trolox/g DW in BC_0_ and a lower value in BV_0_ of 53.94 ± 2.87 mMol Trolox/g DW, suggesting that the betacyanins and polyphenols were the primary compounds responsible for antioxidant activity. The global phytochemical profile of the two varieties studied may be a result of varietal diversity, the influence of vegetation season, as well as climatic and cultivation conditions [26]. 

Regarding the individual betalains, Sawicki et al. [27] studied thirteen varieties and root parts of red beet and identified betanin and isobetanin from betacyanin group and vulgaxanthin I from betaxanthins as the major compounds. These authors reported betaxanthins levels ranging from 2.71 to 4.25 mg/g DW, whereas betacyanins content varied between 8.30 and 13.50 mg/g DW. The scavenging capacity of red beetroot varieties determined by the ABTS assay was found by these authors within the range from 37.68 to 49.71 mol Trolox/g DW, being positively correlated with the total betalain and betacyanin contents. In some other studies, it has been reported that the concentrations of betacyanins and betaxanthins in the roots of red beet varied from 400 and 2100 mg/kg fresh weight and between 200 and 1400 mg/kg fresh weight, respectively [28]. Moreover, the betalain content differs depending on the red beetroot variety [27,29].

### 3.2. The Content of Selected Phytochemicals in Dried Red Beet Purées

It is well known that food drying has as the main purpose the removal of free water from products to a level considered critical for chemical and microbiological reactions while reducing weight and volume, intended to reduce transportation and storage costs [13]. In our study, two drying methods were used for red beetroot purées, namely convective and infrared drying. Following the drying, the phytochemicals profile (Table 1) of the resulting powders were analyzed, to evaluate the losses of the main red beet pigments. A significant decrease in total betalains of approximatively 50% and 43% were observed in BV_C_ and BC_C_, respectively. The infrared technology caused a less intense degradation effect in the total betalains content, with approximatively 50% and 38% in BV_IR_ and BC_IR_, respectively. 

A comparative analysis of the two drying methods on the betalains showed a more protective effect of the infrared technology, with a decrease of approximately 39% in BC_IR_ and 36% in BV_IR_ for betaxanthins, whereas convection drying leads to a significantly higher decrease of approximately 46% for both varieties. The convection drying caused a significant decrease in the betacyanin, with 40% in both varieties, whereas infrared drying leads to a significantly different decrease patterns in the selected purées, with a decrease of 51% in BC_IR_ and approximately 39% in BV_IR_.

Drying by convection of BC_C_ purées caused a significant decrease in polyphenols and flavonoids, of approximately 60% and 52%, respectively. The selected phytochemicals were more stable in BV_C_, with a reduction of approximately 21% and 30%, respectively. However, when drying by infrared technology, polyphenols and flavonoids were found to decrease in BC_IR_ by 62% and 23%, respectively, whereas in BV_IR_ a slight decrease in polyphenols (of 19%) and an increase of 20% in flavonoids content was observed. 

A significant decrease of approximatively 72% was found in antioxidant activity of BC_C_ purées caused by convective drying, whereas heating by infrared of BV_C_ leads to an increase of 70% ABTS radical scavenging activity, due probably to the increase in flavonoid content. The hydrolysis of C-glycosides in the flavonoid composition leads to the formation of monomers that increase the total amount of flavonoids, as well as the antioxidant capacity [30].

Guldiken et al. [31] pointed out that during processing, including drying, the phytochemicals in the red beet products undergo both increases and decrease. For example, Sawicki et al. [32] analyzed three types of processing parameters of red beetroot (boiling, fermentation, and microwave-vacuum treatment) and suggested that the main compound among the betacyanins group in the analyzed products was betanin, while the predominant compound from the betaxanthins group was vulgaxanthin I. This phenomenon occurs as a result of red beet exposure to light and temperature, causing the transformation of betacyanins into decarboxylated forms. A significant reduction of total betalains with approximatively 54% was also observed by these authors when boiling whole roots for 45 min, while the total betacyanin and betaxanthin contents decreased by approximately 43% and 87%, respectively. Similar results were reported by Sawicki and Wiczkowski [33], suggesting a significant 54% reduction in the total betalains content after boiling of whole roots for 60 min.

### 3.3. Cells Viability

During the storage time (28 days at 4 °C), a decrease in the number of *L*. *casei 431*^®^ viable cells of all samples was observed. Thus, the number of *L. casei* 431^®^ cells decreased after 21 days from 8.79 log CFU/g to 7.17 log CFU/g for samples BV_C_ and BV_IR_. For samples BC_C_ and BC_IR_ in the same conditions, the number of *L. casei* 431^®^ cells decreased from 8.58 log CFU/g to 7.89 log CFU/g. A similar tendency was reported by Paraschiv et al. [34] for a probiotic *L. casei* 431^®^ strain, after 21 days, during the storage in refrigeration conditions. The drying method did not significantly affect the cell viability. However, higher viability by 1 log CFU/g was observed in BC purée samples compared with BV purée samples.

For a food to be considered probiotic, it must contain viable probiotic cultures in populations over 6 log CFU/g during the shelf life of the product [35]. *L. casei* 431^®^ remained viable in populations of more than 7 log CFU/g during 21 days of storage for all samples, which makes the red beet purée suitable to be defined as a functional product. 

### 3.4. In Vitro Release of Betalains

**Figure 1:**
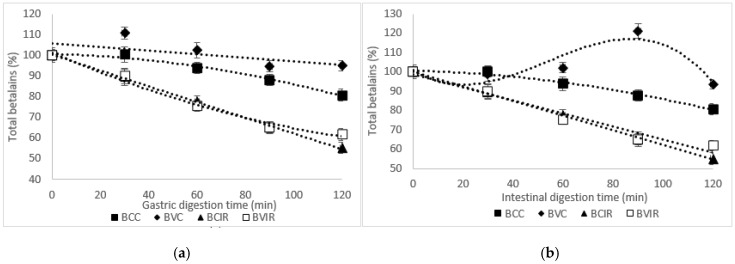
The in vitro digestion of total betalains in powders samples in gastric simulated juice (**a**) and intestinal simulated juice (**b**): BV_C_, BC_C_—red beet purée dried by convection method; BV_IR_, BC_IR_—red beet purée dried by infra-red (IR) method.

The in vitro digestion of purées was determined by simulating the gastric and intestinal phases. The content of the betalains in the digestion phases resulting from in vitro gastrointestinal digestion of red beet products was analyzed. As suggested by Sawicki et al. [32], the obtained results may offer the opportunity to evaluate the changes in the total content of betalains after in vitro digestion and the potential for in vitro bioaccessibility of the main phytochemicals from beetroot dried powders. As shown in Figure 1a, the total betalains content decrease from convection dried red beet powders in BC_C_ after in vitro gastric digestion up to 20% during 120 min of reaction. 

The total concentration of betalains from BV_C_ in the gastric phases were increased by up to 10% after 30 min of digestion and decreased up to a maximum of 5% after 120 min (Figure 1a). Significantly, the decrease in total betalains content continued in intestinal simulated juice for BC_C_, ranging from 6% at the beginning of digestion to a maximum of 19% after 120 min of reaction. In BV_C,_ a release in the total betalains was observed in the first 90 min of intestinal digestion, up to 21%, followed by a decrease in total betalains content (Figure 1b). 

The infrared dried pattern of the total betalains in in vitro digestion is given in Figure 1a. In gastric digestion, BC_IR_ and BV_IR_ showed a significant decrease in total betalains, of approximately 45% and 38%, respectively. In simulated intestinal juice, the decrease reached a maximum value of 23% and 18% BC_IR_ and BV_IR_, respectively, after 120 min of reaction (Figure 1b).

It has been suggested that betacyanins and betaxanthins have broad pH stability in the range pH 3.0–7.0 [36]. However, in a very acidic or basic environments, the molecules are involved in molecular cleavage, decarboxylation, and formation of other products [36]. Therefore, the significant decrease of total betalains in the gastric environment is probably due to the high instability at acidic conditions. Based on our results, a protective effect of the matrices may be considered in intestinal simulated juice. Our results are in good agreement with Sawicki et al. [32] and Tesoriere et al. [37], suggesting the importance of the food matrix in affecting the stability of betalains to the acidic pH.

### 3.5. Structural and Morphological Properties of the Dried Powders

The confocal analysis revealed the presence of many vegetal tissue fragments, coming from beetroot. These fragments present parenchymal cells, with dimensions of 104.91–110.84 µm (for BV sample) and 44.54–116.28 µm (for BC sample). The cellular content is rich in betalains which have an absorption peak at 536–540 nm and a wide emission spectrum, between 506–660 nm [25], and are biologically active compounds responsible for the remarkable antioxidant properties for which red beet is in the top 10 the most valuable vegetables [38]. 

**Figure 2:**
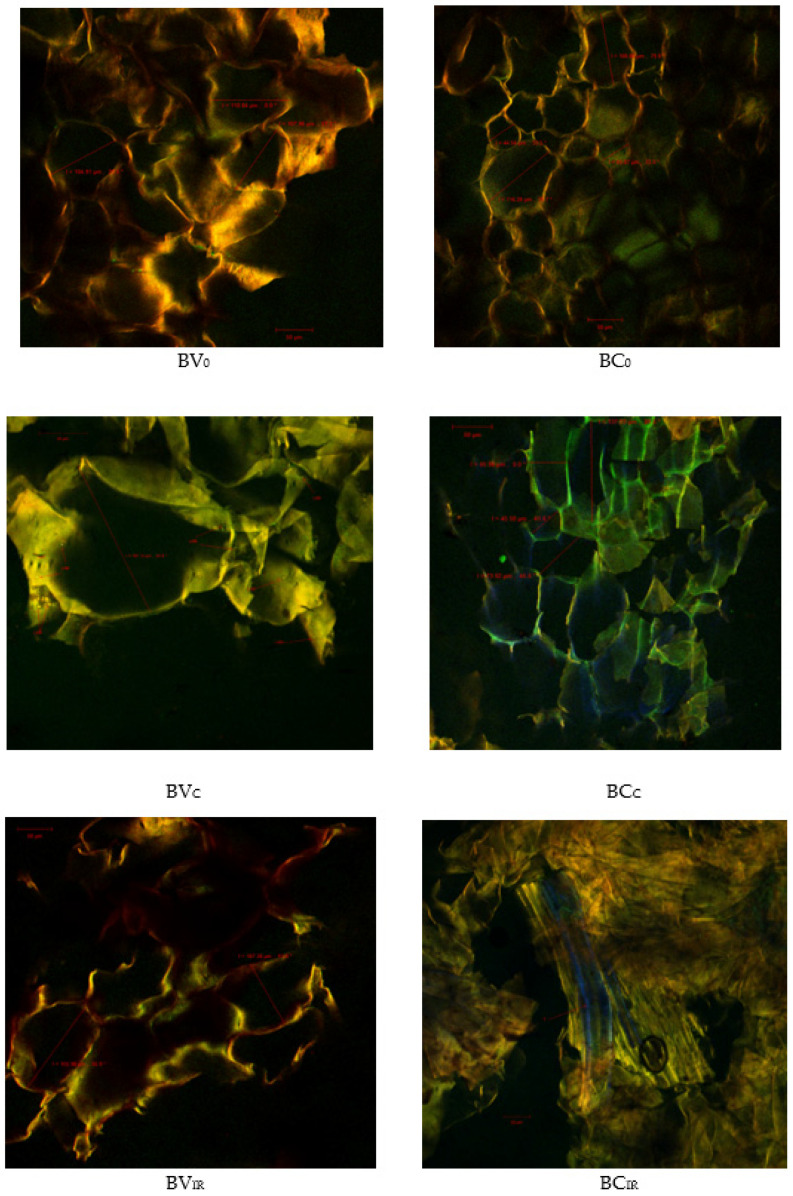
Confocal laser microscopy images of fresh and reconstituted red beetroot purée samples obtained with objective lenses of 20x: BV_0_—fresh sample of *Beta vulgaris* L. var. *vulgaris*; BC_0_—fresh sample of *Beta vulgaris* L. var. *cylindra*; BV_C_, BC_C_—red beet purée dried by convection method; BV_IR_, BC_IR_—red beet purée dried by infra-red (IR) method; *scale bar* 50 μm.

Debris of vascular tissue (fragments of the tracheal elements of xylem), spirally ornamented, are often found, which is an important source of fiber for consumers (Figure 2, BC_IR_). In comparison with the fresh purees, the reconstituted samples coming from IR drying possess a higher number of lysed cells, with fragmented cell walls, as a consequence of rapid dehydration, which also affected the cellular constituents (Figure 2, BV_IR_ and BC_IR_). 

The convection drying technology is milder, a fact proved by the confocal images captured from the reconstituted BV_C_ and BC_C_ samples in which large cells can be visualized after rehydration (187.12 µm, respectively 137.33 µm), with rich content in valuable vegetable pigments (in green-yellow). In the BV_C_ samples, acquired with ZEN 2012 SP1 Black software, zoom 1.2, it can be seen even the microcolonies/biofilms resulting from the aggregation of probiotic lactic acid bacteria (*L. casei* 431 strain) from the functional product. Carbohydrates and biologically active compounds from plant tissue are an excellent support for maintaining the viability of lactic acid bacteria, so they will act synergistically and symbiotically. This result is also supported by previous studies by Barbu et al. [39] who used a strain of *Lb. plantarum* BL3 to obtain three variants of functional products based on beetroot.

### 3.6. FTIR 

**Figure 3:**
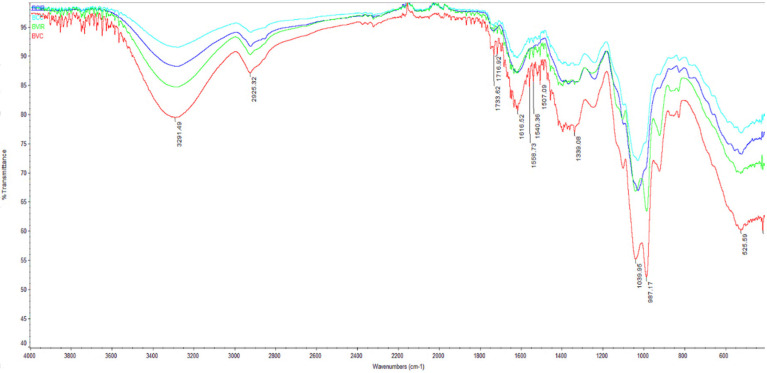
Fourier-transform infrared spectroscopy spectra of red beetroot dried samples: BV_C_, BC_C_—red beet purée dried by convection method; BV_IR_, BC_IR_—red beet purée dried by infra-red (IR) method.

The FTIR spectra of all the red beetroot tested samples were recorded in the spectral range of 400–4000 cm^−1^ are shown in Figure 3. As can be seen, the main differences between the FTIR spectra of convection and IR dried beetroot samples were observed in the spectral ranges of 1028–1039. The FTIR spectra recorded weak to wide bands at 3550 and 1623 cm^−1^, which correspond to stretching vibrations of OH groups and H-O-H bending arising from the moisture content of the red beetroot test samples, respectively. The weak bands were due to the loss of moisture content generated by the drying process. Similar findings were reported by Nesakumar et al. [40] in a study over the spoilage of red beetroot and by Singh et al. [41] for the extraction of betalain pigments from beta Vulgaris peels by microwave pretreatment. 

### 3.7. Color Parameters

According to Henao-Ardila et al. [42] for acceptability of a powdered product, color represents an important parameter considered essential by the consumers, so reconstituted powder should have the same color as the fresh product. A significant effect on the color changes of the red beet is to be found in the processing temperatures and drying methods. The results of the color measurement for fresh and dried red beet purées are shown in Table 2.

The color values (for each type of drying method and plant material) were registered and indicated that the L*, a*, and b* parameters increased for all powder samples as compared to the fresh samples. L* values, which show the whiteness of the product, ranged between 21.16 ± 0.21 and 30.67 ± 0.02 for samples obtained from BV and between 21.19 ± 0.15 and 32.15 ± 0.12 for samples obtained from BC. After drying, the color of powder samples became lighter and reddish probably due to the betacyanins. Ng and Sulaiman [43] observed similar results for beetroot powder while Ochoa-Martinez et al. [44] for beetroot-orange juice. They also reported that thermal processing causes some modifications in red-orange betanins, by increasing the values of the red/green color parameter (a*). The color coordinate (a*) varied between 5.00 ± 0.21 (BV_0_) and 29.14 ± 0.04 (BC_IR_). In the case of dried samples, a maximum change of color was about 51.72% and this percent was established for the sample BC_IR_ compared with the value of the fresh product. The IR drying process may induce rapid heat penetration directly into the product. Therefore, a higher color change in IR dried samples compared with convective dried samples was noticed. b* values of dried red beetroot samples increased compared with the values of the fresh samples. The increase of the b* value was reflected by the decomposition of betanin with the occurrence of yellow compounds as betalamic acid, or condensation of betalamic acid with amino compounds (betaxanthins) or cyclo-DOPA (neobetacianins) [45]. 

The C* values of the fresh red beetroot purée increase after the convection and IR drying process. In general, the C* value ranged from 5.21 ± 0.20 to 30.16 ± 0.25. When the value of the hue angle (h*) presented in Table 2 was checked, it can be concluded that this value tended to grow for all dried samples in the same mode as for the chroma (C*) values. The visual color appearance value (h*) in beetroot powders was the highest in the samples processed by convection method (26.23 ± 0.18 for BV_C_ sample), followed by the fresh sample and the other IR treated samples. The appearance of the product, i.e., the color, is very important for the consumers; for that reason, Δ*E* represents an important color parameter of the dried product, which reveals the human eye’s capacity to differentiate the colors of the samples. The total color change (Δ*E*) of the dried red beetroot samples varied from 8.02 ± 0.27 to 24.37 ± 0.21 units, influenced by the applied drying method and temperature. The L*, a*, and b* parameters were used to appreciate browning index (*BI*), which indicates the purity of the brown color, whiteness index (*WI*), which is an indicator of enzymatic discoloration and yellowness index (*YI*). *YI* is associated with product degradation by light, chemical exposure, and processing. Regarding the BI, the fresh red beetroot purées showed the lowest values (28.57 ± 0.00 for BV_0_ and 35.29 ± 0.00 for BC_0_). In our study, the values of BI increased, due to the enzymatic and non-enzymatic reactions that took place at a low temperature—short period of time. The increased YI values for all dried samples compared to the fresh samples can be assigned to the pigment decomposition and the occurrence of Maillard reactions.

### 3.8. Textural and Oscillatory Measurements 

#### 3.8.1. Texture Analysis 

The texture analysis of the purée samples (Table 3) revealed that IR drying induced fewer damages in the vegetal tissue, comparing with convection drying. This could be concluded from the values of firmness, which are the lowest in the case of fresh pureés (0.76 ± 0.03 N for BV_0_ and 0.72 ± 0.05 N for BC_0_). The rehydrated samples did not manage to absorb the same amount of water which was removed during the drying process. Therefore, the vegetal particles are firmer and tend to gather at the bottom of the container, leading to increased firmness. At the same time, at rehydration, the purée particles did not manage to recover the bonds between them, due to cell war disruptions and fissures as reported by several microstructure studies [12,30]. This fact leads to lower adhesiveness, springiness, and cohesiveness values in the rehydrated samples.

When comparing the values of the textural parameters obtained for convectional drying with those obtained for IR drying, it can be noticed that the latter are closer to fresh samples. It can be concluded that IR drying is milder compared to the convectional one. As for beetroot varieties, from a textural point of view, no major differences between samples were observed.

#### 3.8.2. Rheological Analysis Results

**Figure 4:**
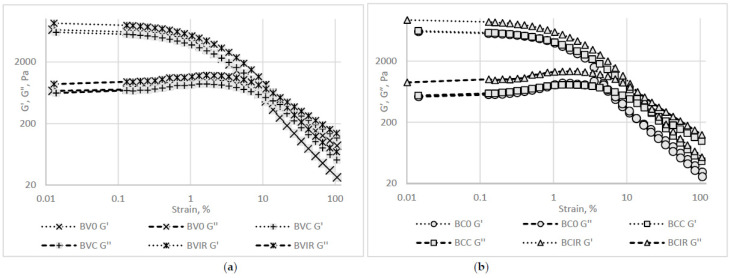
Rheological behavior as revealed by the strain sweep test: (**a**) *Beta vulgaris L. var. vulgaris*, (**b**) *Beta vulgaris L. var. cylindra*.

**Figure 5:**
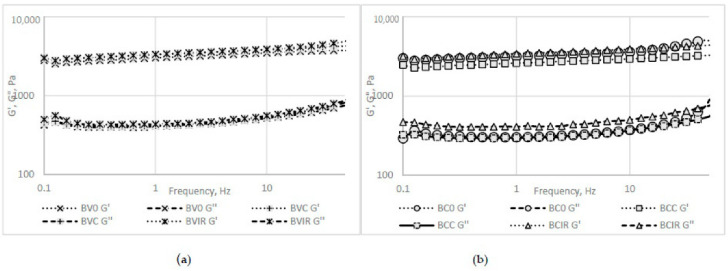
Rheological behavior as revealed by the frequency sweep test: (**a**) *Beta vulgaris L. var. vulgaris*, (**b**) *Beta vulgaris L. var. cylindra*.

The rheological behavior of the fresh and reconstituted beetroot purée samples is presented in Figure 4 and Figure 5. For both of the beetroot varieties purées, the fresh samples registered lower resistance to the applied stress when compared to the reconstituted samples. This behavior could be explained by the insoluble particles presented in the rehydrated samples, which lead to a more rigid network than in the fresh purée, as observed by Leverrier et al. [46] for apple purée. 

The frequency sweep test revealed a gel-like behavior for all purée samples, as the elastic modulus (G′) shows higher values than the loss modulus (G″).

The IR drying determined a reconstituted purée sample that presented the highest consistency, stiffness, and resistance to flow marked by the G′/G″ intersection point, usually associated with the structure breakdown and beginning of the flow.

## 4. Conclusions

Two varieties of red beetroot were studied as potential sources of betalains, polyphenols, and flavonoids in fresh and dried forms of purées, from the perspectives of developing foods or food-grade ingredients with functional values, in terms of phytochemical and lactic acid bacteria. The drying of red beetroot influenced the phytochemical profile of both varieties, with a more protective effect registered for infrared drying processing. However, when drying by infrared technology, polyphenols and flavonoids were found to decrease in *Beta vulgaris* L. var. *cylindra*, whereas in *Beta vulgaris* L. var. *vulgaris*, a slight decrease in polyphenols and an increase in flavonoids content was observed. The decrease in phytochemicals significantly affected the antioxidant activity of the purées, regardless of the drying technology. In all the dried powders, a one logarithmic decrease in lactic acid bacteria viability was registered. After drying, the color of beetroot powder samples became lighter and reddish.

Our study brings some valuable results involving the optimization of drying processes to validate the quality of phytochemicals retention. The high antioxidant activity of red beet powders combined with a 10^7^ CFU/g for lactic acid bacteria represents a good base for the development of a biosynthetic process to obtain additives with a potential for application in the food and pharmaceutical industry.

## Figures and Tables

**Table 1:** Phytochemical profile of fresh and dried red beetroot purées.

Samples Code	Betalains		Total Polyphenols, mg GAE/g DW	Total Flavonoids, mg EC/g DW	ABTS Radical Scavenging Activity, mMol Trolox/g DW
	β-cyanin, mg/g DW	β-xanthin, mg/g DW			
BV_0_	7.03 ± 0.02 ^C^	4.06 ± 0.01 ^C^	32.88 ± 0.34 ^B^	23.31 ± 1.34 ^B,C^	53.94 ± 2.87 ^A^
BC_0_	9.23 ± 0.07 ^C^	4.71 ± 0.06 ^C^	71.94 ± 2.21 ^B^	36.00 ± 1.78 ^B,C^	93.46 ± 2.51 ^A^
BV_C_	4.25 ± 0.02 ^C^	2.53 ± 0.02 ^C^	29.01 ± 0.97 ^B^	16.22 ± 0.81 ^B,C^	26.19 ± 0.32 ^A^
BV_IR_	4.49 ± 0.03 ^C^	2.49 ± 0.02 ^C^	27.76 ± 1.23 ^B^	26.19 ± 1.50 ^B,C^	92.21 ± 0.68 ^A^
BC_C_	4.19 ± 0.02 ^C^	2.21 ± 0.01 ^C^	26.05 ± 0.24 ^B^	14.02 ± 0.49 ^B,C^	55.95 ± 0.57 ^A^
BC_IR_	4.29 ± 0.02 ^C^	2.60 ± 0,08 ^C^	26.47 ± 1.18 ^B^	27.06 ± 1.57 ^B,C^	94.21 ± 0.38 ^A^

BV_0_—fresh sample of *Beta vulgaris* L. var. *vulgaris*; BC_0_—fresh sample of *Beta vulgaris* L. var. *cylindra;* BV_C_, BC_C_—red beet purée dried by convection method; BV_IR_, BC_IR_—red beet purée dried by IR method. Means that do not share a letter as a superscript (A, B, C) are significantly different.

**Table 2:** Effects of drying treatment on color parameters of fresh and dried red beetroot purées.

Color Parameters	Fresh Samples		Dried Samples			
	BV_0_	BC_0_	BV_C_	BV_IR_	BC_C_	BC_IR_
L*	21.16 ± 0.3 ^B^	21.19 ± 0.15 ^B^	25.78 ± 0.15 ^B^	30.67 ± 0.02 ^B^	29.68 ± 0.12 ^B^	32.15 ± 0.12 ^B^
a*	5.00 ± 0.2 ^B,C^	8.32 ± 0.03 ^B,C^	10.29 ± 0.22 ^B,C^	14.19 ± 0.02 ^B,C^	25.66 ± 0.26 ^B,C^	29.14 ± 0.04 ^B,C^
b*	1.45 ± 0.04 ^C^	1.41 ± 0.02 ^C^	5.07 ± 0.21 ^C^	6.56 ± 0.12 ^C^	6.02 ± 0.03 ^C^	7.78 ± 0.04 ^C^
Δ*E*	-	-	8.02 ± 0.27 ^B,C^	14.84 ± 0.28 ^B,C^	19.84 ± 0.28 ^B,C^	24.37 ± 0.21 ^B,C^
C*	5.21 ± 0.20 ^B,C^	8.44 ± 0.03 ^B,C^	11.47 ± 0.24 ^B,C^	15.64 ± 0.03 ^B,C^	26.35 ± 0.04 ^B,C^	30.16 ± 0.25 ^B,C^
h*	16.17 ± 0.31 ^B,C^	9.62 ± 0.11 ^B,C^	26.23 ± 0.18 ^B,C^	24.81 ± 0.47 ^B,C^	13.20 ± 0.06 ^B,C^	14.95 ± 0.10 ^B,C^
*BI*	28.57 ± 0.00 ^A^	35.29 ± 0.00 ^A^	47.05 ± 0.00 ^A^	52.94 ± 0.00 ^A^	76.47 ± 0.00 ^A^	88.23 ± 0.00 ^A^
*WI*	20.98 ± 0.29 ^B,C^	20.74 ± 0.15 ^B,C^	24.89 ± 0.11 ^B,C^	28.93 ± 0.03 ^B,C^	24.90 ± 0.11 ^B,C^	25.75 ± 0.04 ^B,C^
*YI*	9.78 ± 0.28 ^B,C^	9.51 ± 0.09 ^B,C^	28.09 ± 0.28 ^B,C^	30.56 ± 0.60 ^B,C^	28.97 ± 0.13 ^B,C^	34.57 ± 0.22 ^B,C^

BV_0_—fresh sample of *Beta vulgaris* L. var. *vulgaris*; BC_0_—fresh sample of *Beta vulgaris* L. var. *cylindra;* BV_C_, BC_C_—red beet purée dried by convection method; BV_IR_, BC_IR_—red beet purée dried by IR method. Means that do not share a letter as a superscript (A, B, C) are significantly different. L*—clarity; a*—red/green colour component; b*—blue/yellow colour component; Δ*E*—total colour difference; C*—chroma; h*—hue angle; *BI*—browning index; *WI*—whiteness index; *YI*—yellowness index.

**Table 3:** The values of textural parameters.

Parameter	BV_0_	BV_C_	BV_IR_	BC_0_	BC_C_	BC_IR_
Firmness, N	0.76 ± 0.03 ^B^	1.31 ± 0.15 ^B^	0.97 ± 0.04 ^B^	0.72 ± 0.05 ^B^	1.26 ± 0.01 ^B^	1.02 ± 0.04 ^B^
Adhesiveness, mJ	3.38 ± 0.29 ^B^	0.69 ± 0.02 ^B^	0.35 ± 0.01 ^B^	4.58 ± 0.31 ^B^	0.44 ± 0.02 ^B^	0.25 ± 0.02 ^B^
Cohesiveness, -	0.72 ± 0.10 ^B^	0.47 ± 0.01 ^B^	0.46 ± 0.09 ^B^	0.67 ± 0.02 ^B^	0.45 ± 0.01 ^B^	0.42 ± 0.01 ^B^
Springiness, mm	9.18 ± 0.19 ^A^	5.77 ± 0.07 ^A^	6.23 ± 0.10 ^A^	8.72 ± 0.10 ^A^	5.29 ± 0.08 ^A^	6.06 ± 0.07 ^A^

BV_0_—fresh sample of *Beta vulgaris* L. var. *vulgaris*; BC_0_—fresh sample of *Beta vulgaris* L. var. *cylindra*; BV_C_, BC_C_—red beet purée dried by convection method; BV_IR_, BC_IR_—red beet purée dried by infra-red (IR) method. Means that do not share a letter as a superscript (A, B, C) are significantly different.
